# Multidrug-Resistant *Klebsiella pneumoniae* Complex From Clinical Dogs and Cats in China: Molecular Characteristics, Phylogroups, and Hypervirulence-Associated Determinants

**DOI:** 10.3389/fvets.2022.816415

**Published:** 2022-03-10

**Authors:** Zhenbiao Zhang, Liu Zhang, Hegen Dai, Haixia Zhang, Yu Song, Qi An, Jianzhong Wang, Zhaofei Xia

**Affiliations:** ^1^College of Veterinary Medicine, Shanxi Agricultural University, Jinzhong, China; ^2^College of Veterinary Medicine, China Agricultural University, Beijing, China

**Keywords:** *Klebsiella pneumoniae* complex, dog, cat, whole-genome sequence, multidrug resistance

## Abstract

*Klebsiella pneumoniae* complex is an increasingly important bacterial pathogen that is capable of causing severe organs and life-threatening disease. This study aimed to investigate the multidrug resistance, phylogroups, molecular characterization, and hypervirulence-associated determinants of the complex, which were isolated from clinical diseased dogs and cats. A total of 35 *K. pneumoniae* complex (2.3%; 95% confidence interval, 1.6–3.2) isolates were identified from 1,500 samples, all of which were collected randomly from veterinary hospitals in the 12 regions across China. Antimicrobial susceptibility testing showed that isolates were extremely resistant to amoxicillin–clavulanate (82.9%) and trimethoprim–sulfamethoxazole (77.1%). The rate of multidrug-resistant reached an astonishing 82.9% and found a carbapenemase-producing strain carrying IncX3-*bla*_NDM−5_ derived a cat from Zhejiang. The prevalence rates of extended-spectrum β-lactamase gene *bla*_CTX−M_ and plasmid-mediated quinolone resistance gene *aac(6')Ib-cr* were 51.4% and 45.7%, respectively. The resistance gene *aph(3')-Ia* of isolates from cats was more significantly (*p* < 0.05) prevalent than that from dogs. Likewise, *K. pneumoniae* complex harbored hypervirulence-associated genes *ybt* (11.4%), *iuc* (5.7%), and *iroB* (2.9%). Three (8.6%) of the 35 isolates were determined as hypermucoviscous by the string test. Lipopolysaccharide serotype O1v2 had the highest percentage of 25.7%, but capsular serotypes presented diversity distribution among the isolates. The core–genome phylogenetic tree demonstrated most of the isolates belonged to the *KpI* phylogroup (91.4%). Multilocus sequence typing analysis identified 25 different STs; ST15 and ST37 were the most abundant accounting for isolates, followed by ST307, ST656, ST1408, and ST4566. In addition, the prevalence of IncFIB-type plasmid for cat isolates was significantly higher (*p* < 0.05) than that for dogs. Sequences of IncX3 in *bla*_NDM−5_-positive strain contained regions showing >99% nucleotide sequence identity to the reference plasmid pNDM-MGR194 from the human.

## Introduction

*Klebsiella* species, gram-negative opportunistic pathogens, commonly caused acquired antimicrobial-resistant infections in hospitals or communities. They are belonging to the Enterobacteriaceae, which includes *Escherichia, Salmonella*, and *Shigella*. In companion animals, *K. pneumoniae* has been reported to colonize hosts and causes extraintestinal infections, such as urinary tract infections, pyometra, upper respiratory tract infections, and bloodstream infection (septicemia) (1, 2). The treatment of these infections was often difficult because of the emergence of antibiotic resistance, which may be associated with high morbidity and mortality rate (3). In recent years, multidrug-resistant (MDR), carbapenem-resistant *K. pneumonia* (CRKP), and hypervirulent strains (hvKP) spread widely as a critical public health threat in China and even the world (4).

The high incidence of *K. pneumoniae* antimicrobial resistance rate (AMR) received increasing attention, mainly due to the continuous increase in deaths associated with AMR clone produced by CRKP and hvKP. Most of the new AMR genes discovered in the past two decades were first detected and then spread widely among gram-negative bacterial pathogens, including the extended-spectrum β-lactamase (ESBL) forms of *bla*_CTX−M_ and *bla*_SHV_, the carbapenemases *bla*_KPC_ and *bla*_NDM_, and most recently *mcr-1*, the first plasmid-borne gene associated with colistin resistance (5). The emergence of these AMR genes from *K. pneumoniae* not only increases the risk of failure for human antibacterial treatment but also affects that for companion animals. Unfortunately, if the bacteria were transmitted from the pets to their owners, the antimicrobial bacteria from companion animals may have an important impact on human public health (6).

The new technology of molecular strain typing based on DNA sequencing provides various opportunities for elucidating the structure of the *K. pneumoniae* population (7). Multilocus sequence typing (MLST) provided a standardized and replicable system for *K. pneumoniae* identification and naming, which is based on chromosomally encoded seven housekeeping genes (*rpoB, gapA, mdh, pgi, phoE, infB*, and *tonB*) (8). Whole-genome sequencing (WGS) could identify closely related species in clinical and research laboratories that have an average nucleotide homology of 95–96% with *K. pneumoniae* through biochemical or proteomic analysis (9, 10). Sequencing of *wzi* alleles was a marker of capsule serotype (KL), which is highly predictive of capsule (K) serotype and had a strong correlation with KL/K type (11, 12). While O antigen of lipopolysaccharide (LPS) has been defined by sequence identity in the conserved *wzm* and *wzt* genes (13).

The members of the *K. pneumoniae* complex were first distinguished based on the *gyrA* and were designated as the phylogenetic group of *K. pneumoniae*. WGS confirmed that the average nucleotide consistency of the whole genome is ≥3%, which is sufficient to specify new species and help identify other member species: *Klebsiella pneumoniae* (Kp1/*KpI*), *Klebsiella quasipneumoniae* subspecies *quasipneumoniae* (Kp2/*KpIIa*), *K. quasipneumoniae* subspecies *similipneumoniae* (Kp4/*KpIIb*), *Klebsiella variicola* subspecies *variicola* (Kp3/*KpIII*), *K. variicola* subspecies *tropica* (Kp5), *Klebsiella quasivariicola* (Kp6), and *Klebsiella africana* (Kp7) (14, 15). *K. variicola*, an emerging pathogen in humans and had been reported numerous infections worldwide but with a lower frequency in wild and companion animals, can also display the hypermucoviscous (hmKv) and/or hypervirulent (hvKv) phenotypes (16, 17). *K. quasipneumoniae* is a new species discovered in recent years. It has extensive kinship with *K. pneumoniae* and *K. variicola*. *K. quasipneumoniae* is also viscous and easy to acquire resistance and has certain high virulence characteristics (18). In this study, we represented a molecular characterization of *K. pneumoniae* complex isolates collected from diseased companion animals as part of a national surveillance program from different regions in China and examined their epidemiological relatedness.

## Materials and Methods

### Sample Collection

Between November 2017 and October 2019, a total of 1,500 clinical specimens with suspicious bacterial infections were collected from dogs (*n* = 835) and cats (*n* = 665) in veterinary hospitals. These hospitals were distributed in the following 26 regions in China: Guangdong, Shandong, Shanghai, Tianjin, Liaoning, Jiangsu, Hubei, Henan, Hebei, Sichuan, Hunan, Zhejiang, Fujian, Heilongjiang, Inner Mongolia, Shanxi, Shaanxi, Chongqing, Ningxia, Anhui, Jilin, Guangxi, Jiangxi, Hainan, Gansu, and Xinjiang. The sampling sites were not disinfected before collection, and the hosts of all samples came to the veterinary hospital for the first time, or they had been more than 2 months since the last antibiotic administration. In addition, the sampling of companion animals was conducted following the principles of the China Agricultural University Animal Ethics Committee document (no. AW01017102-2). All samples consisted of urine (37.1%, 557/1,500), abscess (14.3%, 215/1,500), skin (8.8%, 132/1,500), ear swabs (5.9%, 88/1,500), nasal swabs (5.4%, 81/1,500), coelomic fluid (5.2%, 78/1,500), throat swabs (4.7%, 71/1,500), surgical infection (4.7%, 70/1,500), tracheal lavage (2.7%, 41/1,500), pyometra (1.2%, 18/1,500), oral swabs (1.2%, 18/1,500), blood (1.1%, 16/1,500), vaginal swabs (0.9%, 14/1,500), eye secretion (0.9%, 13/1,500), and other samples (5.86%, 88/1,500) that contained anal swabs, prostatic fluid, synovial fluid, foreskin swabs, cerebrospinal fluid, and bile.

### Bacterial Isolation and Identification

The collected samples were evenly inoculated on sterile MacConkey inositol adonitol agar (HopeBio, Qingdao, China) containing 100 mg/L carbenicillin and then incubated at 37°C overnight. Clones with the red center were inoculated into a 1-mL volume of brain–heart infusion broth medium (Land Bridge, Beijing, China) and cultured 12 h at 37°C with shaking (200 revolutions/min). DNA of the bacterial solution was extracted by TIANamp Bacteria DNA Kit (Tiangen, Beijing, China) according to the manufacturer's instructions. Subsequently, the DNA was used as the template for polymerase chain reaction amplification of 16S rDNA gene as previously described (19), and amplicons were sequenced to confirm bacterial genus using the BLAST algorithm.

### Antimicrobial Susceptibility Testing

All *K. pneumoniae* complex isolates were subjected to antimicrobial susceptibility testing using the agar/broth dilution method with 16 clinically relevant antibiotics of 10 categories (amoxicillin–clavulanate, piperacillin–tazobactam, ceftazidime–avibactam, cefotaxime, cefepime, meropenem, imipenem, aztreonam, ciprofloxacin, enrofloxacin, gentamicin, amikacin, doxycycline, colistin, florfenicol, and trimethoprim–sulfamethoxazole). *Escherichia coli* ATCC 25922 was used as a quality control organism. Results of minimum inhibitory concentrations (MICs) were explained according to the breakpoints of Clinical and Laboratory Standards Institute documents VET08-ED4:2018 /M100-ED30:2020 and European Commission on Antimicrobial Susceptibility Testing (EUCAST) documents (version 9.0, 2019).

### String Test

String test was conducted to define hypermucoviscous phenotype as previously described (20). All tested strains were cultured on 5% sheep blood agar plates and incubated overnight at 37°C. A standard bacteriological inoculation loop was used to touch the colonies lift gently. A viscous string ≥5 mm in length by stretching bacterial colonies was defined as string test positive.

### WGS and Molecular Analysis

Genomic DNA was extracted from all *K. pneumoniae* complex using TIANamp Bacteria DNA Kit according to the manufacturer's instruction. DNA library was established using KAPA HyperPrep kit (Roche, Basel, Switzerland), and sequencing was performed on the HiSeq X Ten platform (Illumina) with 150-bp paired-end reads by Annoroad Genomics Co., Ltd. The draft genomes were assembled using SPAdes (version 3.9.0) (21). All WGS data for this work were deposited in the GenBank and under BioProject accession no. PRJNA685900. Plasmid Inc types, antibiotic resistance genes, and virulence genes were identified using abricate (https://github.com/tseemann/abricate). Sequence types (STs) of *K. pneumoniae* were determined by the bioinformatics tool at https://cge.cbs.dtu.dk/services/MLST, and *K. variicola* was evaluated through the professional *K. variicola* MLST system (http://mlstkv.insp.mx) (22). Harvest package (version 1.1.2) (23) was used to demonstrate a phylogenetic tree of core–genome alignments for all assembled genomes and visualized using Interactive Tree of Life (http://itol.embl.de/) with the corresponding features of each isolate. Capsule serotype and O antigen were analyzed using Kleborate (https://github.com/katholt/Kleborate). A minimum spanning tree of all STs was generated by BioNumerics version 7.6 (Applied Maths, Belgium) using the BURST algorithm. BLAST Ring Image Generator (24) was used to compare the genetic background of different *bla*_NDM_-carrying plasmids.

### Statistical Analysis

Statistical significance was determined using χ^2^ test and Fisher exact test in SPSS Statistics (version 22; IBM Corporation), and the level of significance was set at *p* < 0.05.

## Results

### Prevalence and Distribution of *K. pneumoniae* Complex

**Figure 1:**
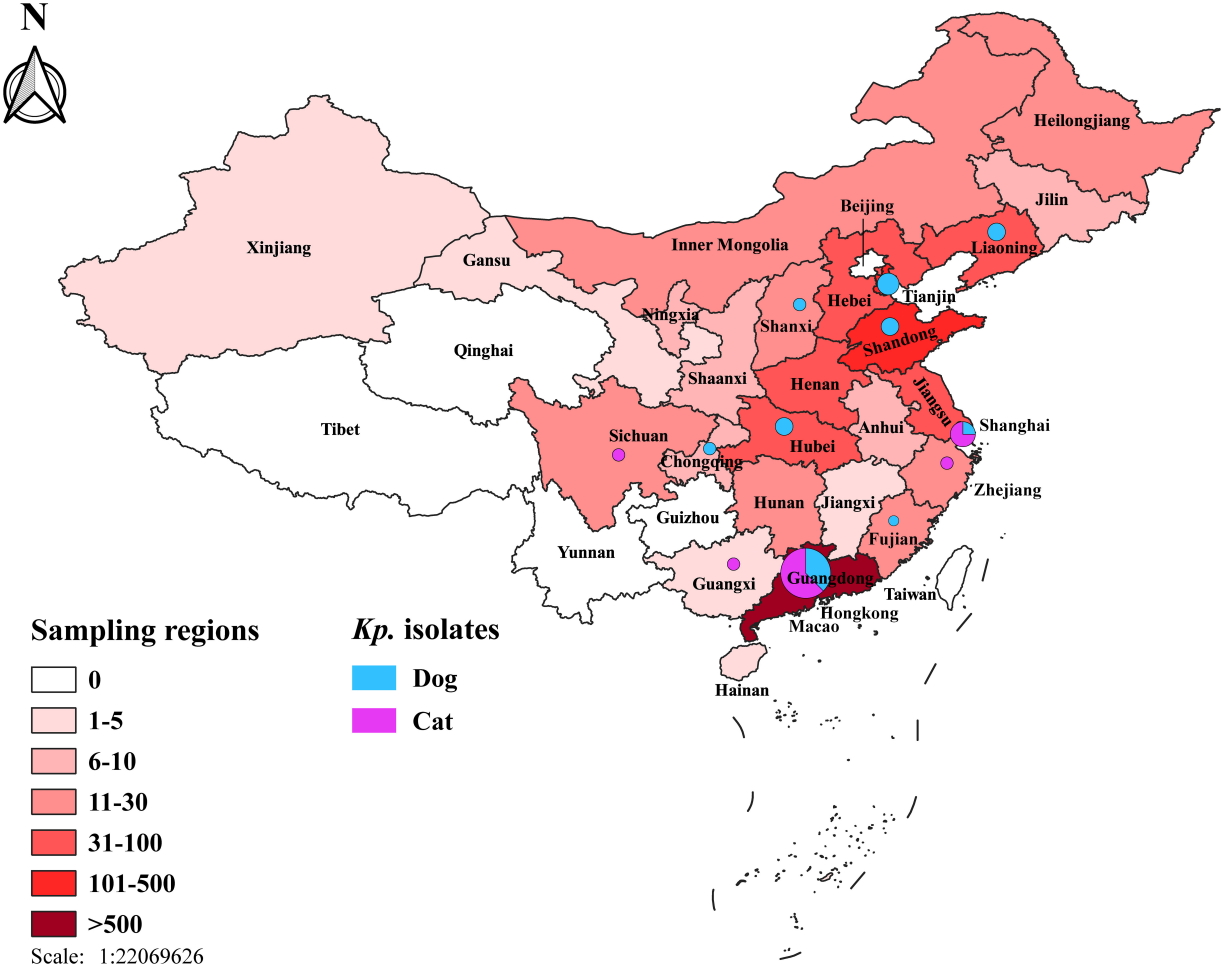
Map of the sampling locations and the prevalence of *K. pneumoniae* complex isolates among dogs and cats in the 26 regions across China. The color gradation stands for the number of samples in different regions. Numbers and the percentages of *K. pneumoniae* complex were demonstrated using isometric pie charts.

Overall, 35 *K. pneumoniae* complex [35 of 1,500; 2.3%; 95% confidence interval (CI), 1.6–3.2] isolates were identified from 1,500 samples, which collected randomly from veterinary hospitals in the 12 regions across China (Figure 1), including 19 from dogs [19 of 835 (2.3%); 95% CI, 1.4–3.5] and 16 from cats [16 of 665 (2.4%); 95% CI, 1.4–3.9]; no significant difference among animals (*p* = 0.868) (Table 1). Two [2 of 35 (0.06%); 95% CI, 0.01–0.19] of *K. variicola* (Kp34 and Kp87) were, respectively, isolated in urine (cat from Shanghai) and skin swab (dog from Shandong). The *K. quasipneumoniae* (Kp36) was obtained from urine of dog from Guangdong (Figure 1). In female companion animals, the isolated rate of *K. pneumoniae* complex from cats was significantly higher than that from dogs (*p* = 0.038). Among samples from cats, most *K. pneumoniae* complex was isolated from urine [8 of 325 (2.5%); 95% CI, 1.1–4.8]. The most strains were isolated from throat swabs of dogs [6 of 56 (10.7%); 95% CI, 4.0–21.9]. Regarding the regions, majority of *K. pneumoniae* complex was isolated from Guangdong (Figure 1), where the largest number of samples was collected. The complex isolates were six of 306 (2.0%; 95% CI, 0.7–4.2) from dogs and 10 of 348 (2.9%; 95% CI, 1.4–5.2) from cats (*p* = 0.451), respectively. No significant difference was found in the prevalence of *K. pneumoniae* complex from different sources and regions between dogs and cats (*p* > 0.05) (Table 1).

**Table 1:** The prevalence and distribution of *K. pneumoniae* complex from dogs and cats.

**Parameters**	**Category**	**Dogs**		**Cats**		***p*-value^**‡**^**
		**No. of samples (%)**	***Kp* isolates (%, 95% CI)**	**No. of samples (%)**	***Kp* isolates (%, 95% CI)**	
Origin	Animals	835 (55.7)	19 (2.3, 1.4–3.5)	665 (44.3)	16 (2.4, 1.4–3.9)	0.868
Gender	Male	475 (56.9)	17 (3.6, 2.1–5.7)	478 (71.9)	10 (2.1, 1.0–3.8)	0.167
	Female	360 (43.1)	2 (0.6, 0.1–2.0)	187 (28.1)	6 (3.2, 1.2–6.9)	0.038^*^
Source	Urine	232 (27.8)	5 (2.2, 0.7–5.0)	325 (48.9)	8 (2.5, 1.1–4.8)	0.813
	Throat swab	56 (6.7)	6 (10.7, 4.0–21.9)	15 (2.3)	1 (6.7, 0.2–31.9)	1.000
	Abscess	138 (16.5)	4 (2.9, 0.8–7.3)	77 (11.6)	1 (1.3, 0–7.0)	0.784
	Nasal cavity	39 (4.7)	0	42 (6.3)	1 (2.4, 0.1–12.6)	1.000
	Ear swab	49 (5.9)	1 (2.0, 0.1–10.9)	39 (5.9)	0	1.000
	Tracheal lavage	30 (3.6)	1 (3.3, 0.1–17.2)	11 (1.7)	0	1.000
	Coelomic fluid	34 (4.1)	0	44 (6.6)	1 (2.3, 0.1–12.0)	1.000
	Skin	97 (11.6)	2 (2.1, 0.3–7.3)	35 (5.3)	2 (5.7, 0.7–19.2)	0.613
	Surgical infection	50 (6.0)	0	20 (3.0)	1 (5.0, 0.1–24.9)	0.286
	Eye secretion	7 (0.8)	0	6 (0.9)	1 (16.7, 0.4–64.1)	0.462
	Other specimens	103 (12.3)	0	51 (7.7)	0	—
Regions	Guangdong	306 (36.6)	6 (2.0, 0.7–4.2)	348 (52.3)	10 (2.9, 1.4–5.2)	0.451
	Shanghai	52 (6.2)	1 (1.9, 0–10.3)	84 (12.6)	3 (3.6, 0.7–10.1)	0.975
	Tianjin	87 (10.4)	3 (3.4, 0.7–9.7)	40 (6.0)	0	0.551
	Hubei	23 (2.8)	2 (8.7, 1.1–28.0)	13 (2.0)	0	0.525
	Liaoning	43 (5.1)	2 (4.7, 0.6–15.8)	17 (2.6)	0	1.000
	Shandong	114 (13.7)	2 (1.8, 0.2–6.2)	54 (8.1)	0	1.000
	Fujian	7 (0.8)	1 (14.3, 0.4–57.9)	15 (2.3)	0	0.318
	Guangxi	2 (0.2)	0	3 (0.5)	1 (33.3, 0.8–90.6)	1.000
	Shanxi	14 (1.7)	1 (7.1, 0.2–33.9)	2 (0.3)	0	1.000
	Sichuan	15 (1.8)	0	13 (2.0)	1 (7.7, 0.2–36.0)	0.464
	Zhejiang	17 (2.0)	0	6 (0.9)	1 (16.7, 0.4–64.1)	0.261
	Chongqing	6 (0.7)	1 (16.7, 0.4–64.1)	2 (0.3)	0	1.000
	Other regions	149 (17.8)	0	68 (10.2)	0	—

*Data is the prevalence and distribution of samples and K. pneumoniae complex from companion animals. The p-values are for comparisons of K. pneumoniae complex between dogs and cats*.

‡ *p-values were determined by Chi-square (χ^2^) and Fisher's exact test in SPSS Statistics*.

* *p < 0.05*.

^**^p < 0.01.

^—^, *no data*.

### Antimicrobial Susceptibility and Resistance

Of these *K. pneumoniae* complex from companion animals, resistance detection rate for dogs (1.9%; 95% CI, 1.1–3.1; *n* = 16/835) was not statistically different from the rate of cats (2.4%; 95% CI, 1.4–3.9; *n* = 16/665) (*p* = 0.514). Antimicrobial susceptibility testing of the 35 isolates showed that they were extremely resistant to amoxicillin–clavulanate (82.9%, *n* = 29/35) and trimethoprim–sulfamethoxazole (77.1%, *n* = 27/35) (Table 2). There were 29 isolates resistant to more than three categories of antibiotics; the rate of MDR reached an astonishing 82.9%, and the MDR rate was no different between dogs and cats (*p* = 0.187). MIC_50_ and MIC_90_ of strains from cats were generally greater than or equal to dogs. Among them, the MIC_50_ of amoxicillin and that of cefepime to cat strains were twice that of dog strains; cefotaxime and amikacin were four times, and aztreonam and enrofloxacin were 32 times. However, the florfenicol-MIC_50_ of dog isolates was 16 times higher than that of cats. Besides, the MIC_90_ values of ciprofloxacin and amoxicillin–clavulanate of strains from cats were two and four times higher than those of dog strains, respectively. *K. pneumoniae* complex from cats showed higher resistance frequencies against all antibiotics than that from dogs (*p* > 0.05) (Table 2). Unfortunately, there was one CRKP isolated from a cat in Zhejiang, which was concurrently resistant to all tested antibiotics except aztreonam and colistin. Also, two strains were resistant (MIC = 32 mg/L and MIC = 8 mg/L) to colistin according to EUCAST.

**Table 2:** Antimicrobial resistance of clinical *K. pneumoniae* complex isolates from dogs and cats.

**Antimicrobial agents**	**All isolates (*n* = 35)**	**Dog (*****n*** **=** **19)**			**Cat (*****n*** **=** **16)**			***p*-value^**‡**^**
	**Resistance, %**	**MIC_**50**_**	**MIC_**90**_**	**Resistance, %**	**MIC_**50**_**	**MIC_**90**_**	**Resistance, %**	
Amoxicillin-clavulanate	29 (82.9)	32/16	64/32	14 (73.7)	64/32	>256/128	15 (93.8)	0.187
Piperacillin-tazobactam	14 (40.0)	4/4	>256/4	5 (26.3)	32/4	>256/4	9 (56.3)	0.094
Ceftazidime-avibactam	1 (2.9)	0.25/4	0.5/4	0	0.25/4	1/4	1 (6.3)	0.457
Cefotaxime	22 (62.9)	64	>256	10 (52.6)	256	>256	12 (75.0)	0.293
Cefepime	17 (48.6)	4	>256	9 (47.4)	8	256	8 (50.0)	1.000
Meropenem	1 (2.9)	0.06	0.125	0	0.06	0.125	1 (6.3)	0.457
Imipenem	1 (2.9)	0.5	1	0	0.5	1	1 (6.3)	0.457
Aztreonam	20 (57.1)	4	>256	9 (47.4)	128	>256	11 (68.8)	0.306
Ciprofloxacin	22 (62.9)	2	128	10 (52.6)	32	256	12 (75.0)	0.293
Enrofloxacin	21 (60.0)	1	64	9 (47.4)	32	64	12 (75.0)	0.166
Gentamicin	22 (62.9)	64	>256	11 (57.9)	64	>256	11 (68.8)	0.727
Amikacin	11 (31.4)	2	>256	4 (21.1)	8	>256	7 (43.8)	0.273
Doxycycline	23 (65.7)	32	64	11 (57.9)	32	64	12 (75.0)	0.476
Colistin	2 (5.7)	2	2	0	2	2	2 (12.5)	0.202
Florfenicol	23 (65.7)	256	>256	13 (68.4)	16	256	10 (62.5)	0.736
Trimethoprim-sulfamethoxazole	27 (77.1)	>32/608	>32/608	14 (73.7)	>32/608	>32/608	13 (81.3)	0.700

‡ *p-values were determined by Fisher's exact test in SPSS Statistics*.

^*^p < 0.05.

^**^p < 0.01.

### Genotypes of Resistance and Virulence

**Figure 2:**
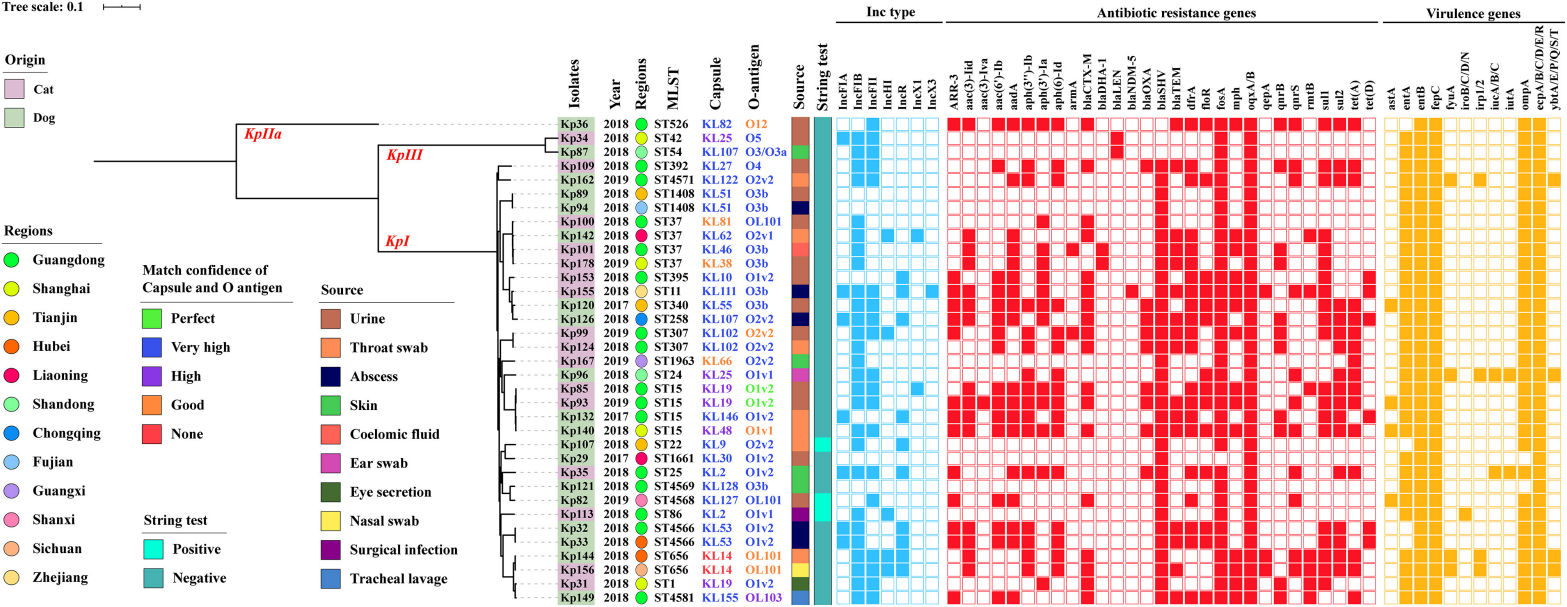
Core genome-based phylogenetic tree and distribution of *K. pneumoniae* complex phylogroups, origins, isolated years, collected regions, MLST, capsular serotype, LPS O antigen, isolate sources, string test, Inc-type plasmids, antibiotic resistance genes, and virulence genes among isolates from companion animals. The origins, regions, KL/O match confidence, sources, and string test of *K. pneumoniae* complex are differentiated by color. Inc-type plasmids, antibiotic resistance genes, and virulence genes are denoted by filled squares for the presence and empty squares for absence.

Carbapenem resistance gene *bla*_NDM−5_ was harbored in one isolate of abscess from cat in Zhejiang, but it was not detected in dogs, and other *bla*_NDM_ variants were not found (Figure 2). The *bla*_SHV_ (91.4%, *n* = 32/35) were the most prevalent resistant genes in companion animals. ESBL gene *bla*_CTX−M_ was the second commonly present in dogs (22.9%, *n* = 8/35) and cats (28.6%, *n* = 10/35), respectively (*p* = 0.315). There were seven CTX-M genotypes (−3, −14, −15, −27, −55, −65, −122), dominated by *bla*_CTX−M−15_ (22.9%, *n* = 8/35) and *bla*_CTX−M−55_ (10.3%, *n* = 4/35). There was one strain with coexisting *bla*_CTX−M−55_ and *bla*_CTX−M−122_, which were isolated from tracheal lavage of a dog in Guangdong. The other ESBL and AmpC-containing isolates harbored *bla*_OXA_ (25.7%, *n* = 9/35) and *bla*_DHA_ (5.7%, *n* = 2/35). The *bla*_LEN_ (*n* = 2) and *bla*_OKP_ (*n* = 1) were only harbored by *K. variicola* (Kp34 and Kp87) and *K. quasipneumoniae* (Kp36). Aminoglycoside-non-susceptible isolates frequently harbored *aph(3*″*)-Ib* (45.7%, *n* = 16/35) and along with *aph(6)-Id*. The resistance gene *aph(3')-Ia* of isolates from cats was more significantly (*p* = 0.042) prevalent than that from dogs. Among the assessed plasmid-mediated quinolone resistance genes, *aac(6')Ib-cr, qnrB, qnrS*, and *oqxAB* were 16 (45.7%, *n* = 16/35), 11 (31.4%, *n* = 11/35), 11 (31.4%, *n* = 11/35), and 35 (100%, *n* = 35/35), respectively. The prevalence of other resistance genes that we obtained was also severely resistant gene; *fosA, dfrA*, and *sul1* were prevalent in isolates (Figure 2).

In addition, *K. pneumoniae* complex harbored 11.4% (*n* = 4/35) yersiniabactin (*ybtA/E/P/Q/S/T*), 5.7% (*n* = 2/35) aerobactin (*iucA/B*), and 2.9% (*n* = 1/35) salmochelin (*iroB/C/D/N*), but other key virulence genes such as colibactin (*clb*) and regulators of mucoid phenotype genes (*rmpA*/*A2*) were not detected. Gene *ybt* and *iuc* coexisted in strain Kp96 (ST24), which was from an ear swab of a dog in Shandong (Figure 2). Besides, virulence-associated genes such as enterotoxins (*astA, entA/B*), ferrienterochelin receptor (*fepC*), yersiniabactin receptor (*fyuA*), yersiniabactin biosynthesis (*irp1/2*), aerobactin receptor (*iutA*), outer membrane protein (*ompA*), and common pili (*ecpA/B/C/D/E/R*) were identified using abricate and demonstrated in Figure 2, and there was no significant difference in virulence-associated genes between dogs and cats (*p* > 0.05).

### Hypermucoviscosity, Capsule Serotype, and O Antigen of *K. pneumonia* Complex

**Figure 3:**
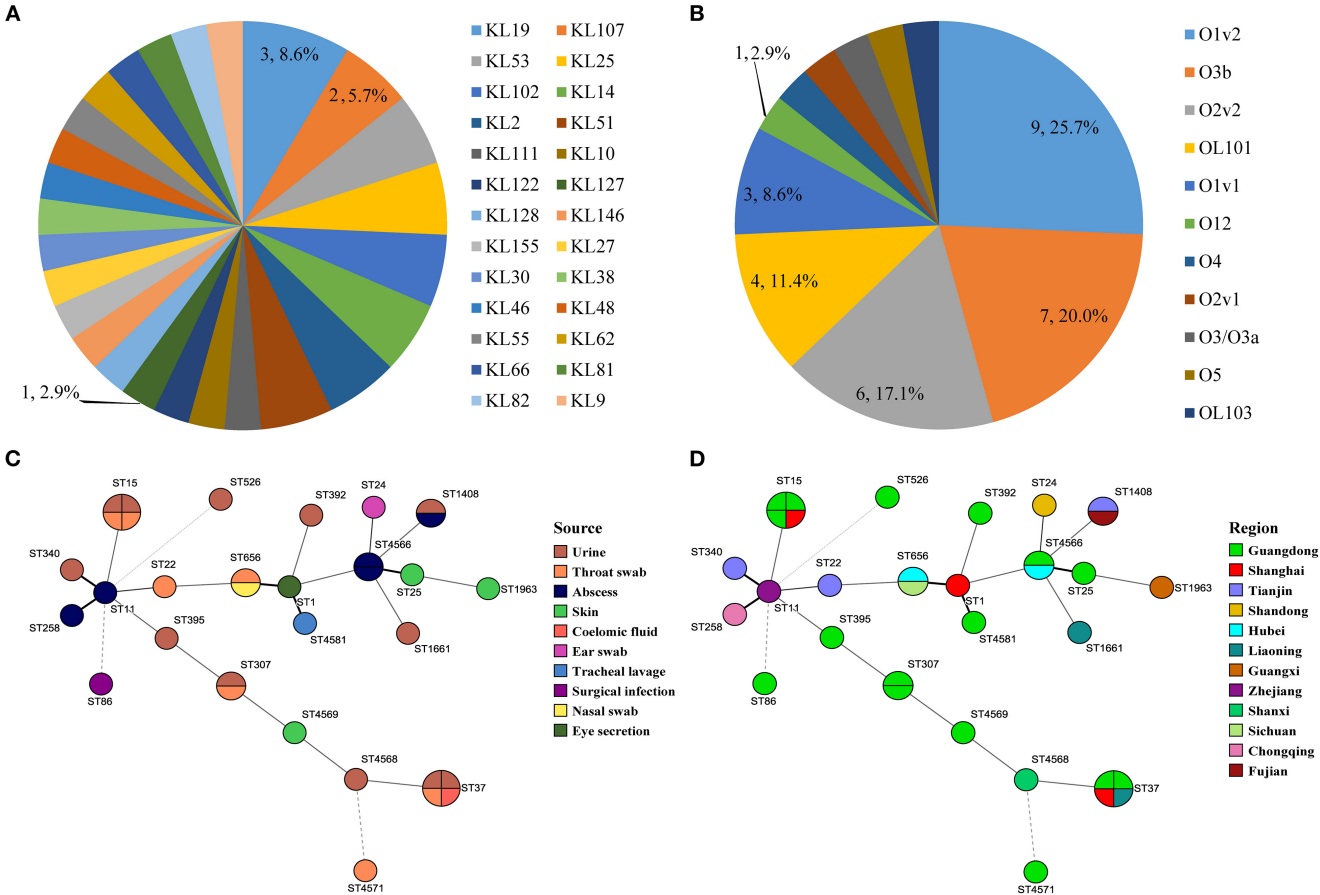
Serotype distribution of capsular serotype and O antigen **(A,B)**. Minimum spanning trees of MLST data (except *K. variicola*) based on source and region **(C,D)**.

Three of the 35 isolates (8.6%) were determined to be hypermucoviscous *K. pneumonia* (hmKp) by the string test. One of the hmKp that indicated strong virulence had a KL2 capsular serotype and O1v1 LPS serotype, and the remaining two strains were serotype KL9/O2v2 and KL127/OL101 (Figure 2). Isolates covered 26 capsular serotypes and presented diversity distribution. KL19 was the most abundant (8.6%, *n* = 3/35), accounting for three isolates, followed by KL2, KL25, KL51, KL53, KL102, and KL107, and two *K. pneumonia* strains with KL14 but match confidence was none (Figures 2, 3A). While O1v2 had the highest percentage of 25.7% (*n* = 9/35) and then O3b, O2v2, O1v1, and OL101 (Figure 3B). The O antigens of three-string test–positive isolate were O1v1, O2v2, and OL101, respectively. O antigen named OL101 onward was defined on the basis of gene content and was not yet associated with a specific serologically defined O type.

### MLST and Phylogenetic Analysis

Core–genome phylogenetic tree demonstrated most of the isolates belonged to *KpI* phylogroup (91.4%, *n* = 32/35), with only one being *KpIIa* and two appertained to the *KpIII* phylogroup (Figure 2). MLST analysis identified 25 different STs among the 35 *K. pneumoniae* complex; the diversity was similar to the capsular serotypes, and five of them were novel STs (ST4566, ST4568, ST4569, ST4571, ST4581). ST15 and ST37 (11.4%, *n* = 4/35) were the most abundant accounting for isolates and followed by ST307, ST656, ST1408, and ST4566. The STs of *K. variicola* Kp34 and Kp87 were ST42 and ST54; they were from the urine of cat and a skin swab of dog, respectively (Figure 2). ST of *bla*_NDM−5_-positive *K. pneumoniae* was ST11. Apart from the *KpIIa* phylogroup, each lineage comprised strains from dogs and cats. Minimum spanning tree analysis further supported the commonality of *K. pneumoniae* complex from different sources and regions with the same STs (Figures 3C,D).

### Plasmid Profiles and Genetic Context of *bla*_NDM_

**Figure 4:**
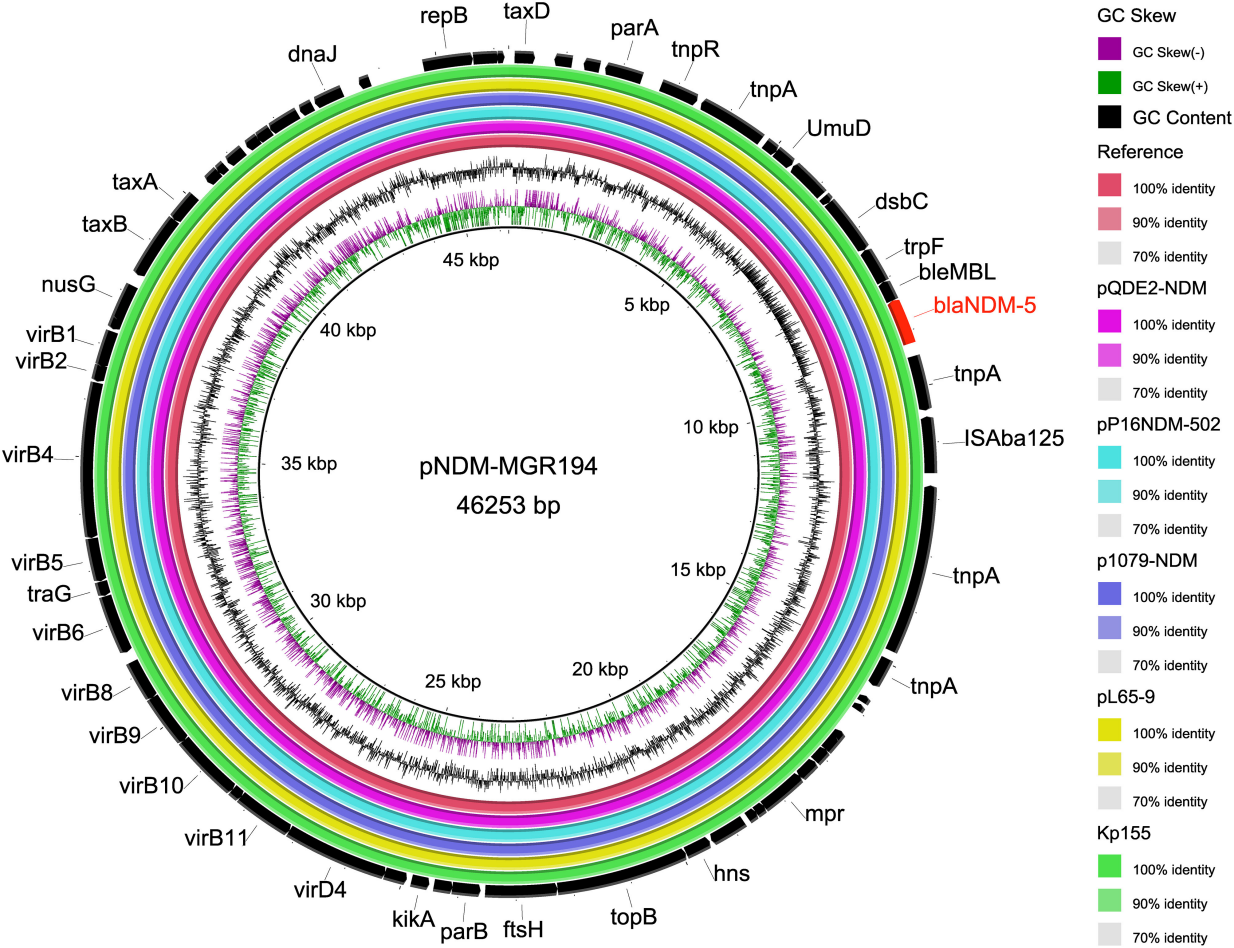
Comparison of whole-genome sequences of plasmid IncX3 carrying *bla*_NDM−5_. Each ring represents an isolated strain. The internal ring is the reference sequence of NDM-5 carrying plasmid pNDM-MGR194 (46253bp, GenBank accession no. KF220657), and the outside rings are the other five plasmids, including Kp155 from this study. From inside to outside, the rings were derived from human (pQDE2-NDM, MH917280), dog (pP16NDM-502, MN701974), chicken (p1079-NDM, MG825384), and goose (pL65-9, CP034744).

The backbone sequences were assembled, and all contigs and gaps were identified by WGS analyses. Through comparison and monitoring, we obtained seven Inc-type plasmids, which were dominant by IncFIB (77.1%, *n* = 27/35), IncFII (54.3%, *n* = 19/35), IncR (28.6%, *n* = 10/35), followed by IncFIA (20%, *n* = 7/35), IncHI (14.3%, *n* = 5/35), IncX1 (5.7%, *n* = 2/35), and IncX3 (2.9%, *n* = 1/35) (Figure 2). The prevalence of IncFIB plasmid for cat isolates was significantly higher than that for dogs (*p* = 0.047). In addition, BLASTn results demonstrated that *bla*_NDM−5_ was harbored on IncX3-type in plasmid *bla*_NDM−5_-positive strains (Kp155), which also contains IncFIA, IncFIB, IncHI, and IncR (Figure 2). Sequences of IncX3 in Kp155 (ST11), which is a source of abscess derived from cat in Zhejiang, contained regions showing >99% nucleotide sequence identity to the reference plasmid pNDM-MGR194 (46253bp, GenBank accession no. KF220657). And *bla*_NDM−5_ was included in an insertion sequence (IS) cassette (ΔIS*Aba125*-IS*5*-*bla*_NDM_-*ble*-*trpF*-*dsbC*-IS*26*) compared by ISfinder, which was consistent with *bla*_NDM−5_-carrying plasmids originating from human (pQDE2-NDM, MH917280), dog (pP16NDM-502, MN701974), chicken (p1079-NDM, MG825384), and goose (pL65-9, CP034744) (Figure 4).

## Discussion

China Antimicrobial Surveillance Network (CHINET) has well-documented the antimicrobial resistance of humans in China (http://www.chinets.com/). However, investigations on companion animals are still lacking in this regard in China. In the current study, we collected 1,500 clinical samples of companion animals for the isolation of *K. pneumoniae* complex and investigated the prevalence of antibiotic resistance, virulence, and molecular typing through whole-genome analysis. Thirty-five *K. pneumoniae* (2.3%) complex was identified from 1,500 samples, which was a lower isolation rate compared with Italy (3.53%) (25). There was no significant difference among animals, 19 from dogs and 16 from cats; the isolation rate of *K. pneumoniae* complex from cats was significantly higher than that from dogs in female animals. But the rate of MDR, 82.9%, was higher than that reported in Singapore (50%) (26). MIC_50_ and MIC_90_ of antibiotics to *K. pneumoniae* complex from cats were generally greater than or equal to that from dogs, suggesting that the resistance of feline strains was more severe than that of dogs. The results were consistent with previous investigations in Iberian Peninsula (27) and China (28). *K. pneumoniae* complex from cats showed no significant difference (*p* > 0.05) against all antibiotics compared with that from dogs, which was consistent with the previous study in South Korea (29). In our study, the resistance of amoxicillin–clavulanate and trimethoprim–sulfamethoxazole were 82.9% and 77.1%, all generally higher than those reported in Portugal (30). One CRKP was resistant to meropenem and imipenem and harbored carbapenemase gene *bla*_NDM−5_, which has been reported in humans (31) and other animals (32), but rarely detected in companion animals.

We found a higher prevalence of ESBLs in *K. pneumoniae* complex clinical isolates (57.1%, *n* = 20/35), compared with those from companion animals in Japan (34.8%) (1), Italy (21.4%) (25), Germany, and other European countries (7.6%) (33). In this study, ESBLs were CTX-M-genotypes (−3, −14, −15, −27, −55, −65, −122) and SHV-genotypes (−41, −42), which was not quite the same as that in previous reports (34). Among CTX-M genotypes, which were dominant by *bla*_CTX−M−15_ (22.9%, *n* = 8/35) and *bla*_CTX−M−55_ (10.3%, *n* = 4/35), there was one strain with coexisting *bla*_CTX−M−55_ and *bla*_CTX−M−122_. In particular, the CTX-M ESBL genes were widely present in the field of human medicine, and the CTX-M-15–producing *K. pneumoniae* complex was the most frequently detected genotype associated with extended-spectrum antibiotic resistance in humans and animals (35). Because of the close contact between companion animals and humans, the genotypes of *K. pneumoniae* complex from companion animals in this study were similar to those of humans in China. Otherwise, *bla*_SHV_ (91.4%) was the most prevalent resistant genes, which were constituted by SHV-187 (28.6%, *n* = 10/35), −106 (17.1%, *n* = 6/35), −110 (14.3%, *n* = 5/35), −182 (11.4%, *n* = 4/35), −145 (5.7%, *n* = 2/35), and SHV-11,−28,−41,−42, and−62 (each 2.9%, 1/35) in our study. The other ESBL and AmpC-containing isolates harbored *bla*_OXA_ (25.7%, *n* = 9/35) and *bla*_DHA_ (5.7%, *n* = 2/35). The *bla*_LEN_ (*n* = 2) and *bla*_OKP_ (*n* = 1) were only harbored by *K. variicola* (Kp34 and Kp87) and *K. quasipneumoniae* (Kp36). *K. variicola* has been widely recognized as an important opportunistic human pathogen commonly involved in hospital-acquired infections; multiple antibiotic resistance genes have been shown to exist, such as clinically relevant resistance determinants such as *bla*_CTX−M_, *bla*_DHA_, and *bla*_LEN_. The *bla*_LEN_ gene corresponds to an intrinsic chromosomal β-lactamase in the *K. variicola* genome (36). Meanwhile, *bla*_DHA_ has been indicated to cause the resistance of *K. pneumoniae* complex in companion animals from a veterinary hospital in Switzerland (37). Population diversity studies have shown that *K. pneumoniae* is phylogenetically closely related to *K. variicola* and *K. quasipneumoniae* (38). The *bla*_OKP_ β-lactamases, closely related to *bla*_SHV_, and the OKP type enzyme were also clearly found in the phylogenetic group *KpII* of *K. quasipneumoniae* (39). Some variant *K. variicola* has been identified from multiple sources, including environments; humans; animals such as dogs, birds, monkeys, and cattle (mastitis); and plants such as coriander (food supply). As potential pathogens of zoonotic, *K. variicola* from companion animals could be transmitted to humans (16).

Hypervirulence and hypermucoviscosity are two different *K. pneumoniae* phenotypes; they could predict positive value by the string test and molecular markers such as the virulence genes *ybt, clb, iuc, iro, rmpA*, and *rmpA2* (40). Three of (8.6%, *n* = 3/35) the 35 isolates were determined to be hypermucoviscous by string test in our study. Kleborate is a tool to screen genome assemblies of *K. pneumoniae* complex and the complex for integrative conjugative element–associated virulence loci (*ybt, clb*) and virulence plasmid–associated loci (*iro, iuc, rmpA, rmpA2*) (41). In this study, one isolate of ST24 harbored *ybt* and *iuc*, which derived an ear swab from a dog in Shandong, and its virulence gene score was evaluated by Kleborate as four. Also, one other strain scored 3 because it harbored *iuc*, and three isolates appeared *ybt*, so it was scored 1. The capsular polysaccharide is located outside the outer membrane; the most common hvKp capsule types are K1, K2, K5, K20, K54, and K57, of which K1 and K2 account for ~70% of hvKp isolates. Otherwise, hvKp strain also has the O antigen, which is part of LPS. K1 and K2 capsule types are usually related to the O1 O antigen type (42). In the current study, capsular presented diversity distribution and covered 26 serotypes, which were predominated by KL19. One of the hmKp that indicated strong virulence had a KL2 capsular serotype and O1v1 LPS serotype. While LPS serotype O1v2 had the highest percentage of 25.7%, the O antigens of three-string test–positive isolate were O1v1, O2v2, and OL101, respectively.

Global problem clones have been isolated from a series of animals, such as ST11 in poultry, ST15 in companion animals, ST23 in non-human primates and horses, and ST25 in pigs (15). Not surprisingly, ST15 and ST37 (11.4%, *n* = 4/35) were the most abundant accounting for isolates in this study. Previous researches on dairy farms in the United States (43), canals or farms in Thailand (44), and farms in the North of England (45) had revealed a large amount of diversity between clinical isolates, and non-human samples. MLST analysis identified 25 different STs among the 35 *K. pneumoniae* complexes in our research, which diversity was similar to the capsular serotypes, and five of them were novel STs (ST4566, ST4568, ST4569, ST4571, ST4581). The STs of *K. variicola* Kp34 and Kp87 from companion animals were ST42 and ST54, respectively. ST42 was mainly prevalent in humans of Mexico; ST54 was dominantly found in the United States and Vietnam according to previous research (22). In China, *K. variicola* from both humans and plants has been described, with ST65 and ST92 corresponding to human isolates described in different reports (22). However, the report of *K. variicola* from the source of companion animals is the first. CRKP or MDR ST11 *K. pneumoniae* harboring KL64 or KL47 and virulence plasmids carrying *iuc* (or *rmpA2*) is widely spread in China (46). Coincidentally, the ST of plasmid IncX3-*bla*_NDM−5_-positive and MDR *K. pneumoniae* was ST11 in our results. Otherwise, some researchers believe that hvKP could accumulate plasmids carrying virulence and resistance genes, which can continuously enhance its resistance to major antibiotics (47). Inc-type plasmids were analyzed and suggested that there was some correlation between MDR and plasmid type. These findings indicated that the variants of *K. pneumoniae* complex resistance genes, virulence genes, and mobile plasmid elements are not limited to certain hosts, emphasizing the need for coordinated control in the concept of One Health.

## Conclusion

We found a high prevalence of MDR *K. pneumoniae* complex isolates from sick dogs and cats in different regions of China. The abuse of combination medications was likely contributed to that, and especially widespread use of amoxicillin–clavulanate and trimethoprim–sulfamethoxazole in the veterinary hospital. These strains harbored *bla*_SHV_, *bla*_CTX−M_, and *bla*_NDM_, and most promoted an MDR profile. Meanwhile, the emergence of hvKP and epidemic clones has increased the risks of veterinarians. Diversity analysis of the core–genomes and STs of this clinical *K. pneumoniae* complex from different sources and regions suggested that the dissemination of Inc-type plasmids has broad reservoirs in *K. pneumoniae* complex. Relevant measures must be formulated to suppress or block transmission of high-risk *K. pneumoniae* complex clonal lineages to ensure the safety of companion animal practitioners and public health.

## Data Availability Statement

The datasets presented in this study can be found in online repositories. The names of the repository/repositories and accession number(s) can be found at: https://www.ncbi.nlm.nih.gov/, PRJNA685900.

## Ethics Statement

The animal study was reviewed and approved by China Agricultural University Animal Ethics Committee document (No. AW01017102-2). Written informed consent was obtained from the owners for the participation of their animals in this study.

## Author Contributions

ZX and JW: conceived and designed study, collected, complied, and analyzed data. LZ, HD, and HZ: statistical analyses. YS and QA: collected the clinical samples. ZZ and JW: drafted and edited manuscript. All authors contributed to the article and approved the submitted version.

## Funding

This work was supported by the Beijing Science and Technology Planning Project (Z171100001517008), Project of Science and Technology Innovation Fund of Shanxi Agricultural University (2021BQ70 and 2021BQ06), Central Funds Guiding the Local Science and Technology Development In Shanxi Province (YDZJSX2021A034), Fund Program for the Scientific Activities of Selected Returned Overseas Professionals in Shanxi Province (20210012), Project of Scientific Research for Excellent Doctors, Shanxi Province, China (SXBYKY2021047), and Research Fund (Clinical Diagnosis and Treatment of Pet) for Young College Teachers in Ruipeng Commonweal Foundation (RPJJ2020021).

## Conflict of Interest

The authors declare that the research was conducted in the absence of any commercial or financial relationships that could be construed as a potential conflict of interest.

## Publisher's Note

All claims expressed in this article are solely those of the authors and do not necessarily represent those of their affiliated organizations, or those of the publisher, the editors and the reviewers. Any product that may be evaluated in this article, or claim that may be made by its manufacturer, is not guaranteed or endorsed by the publisher.
